# Diagnostic Performance of Fully Automated Pixel-Wise Quantitative Myocardial Perfusion Imaging by Cardiovascular Magnetic Resonance

**DOI:** 10.1016/j.jcmg.2018.01.005

**Published:** 2018-02-14

**Authors:** Li-Yueh Hsu, Matthew Jacobs, Mitchel Benovoy, Allison D. Ta, Hannah M. Conn, Susanne Winkler, Anders M. Greve, Marcus Y. Chen, Sujata M. Shanbhag, W. Patricia Bandettini, Andrew E. Arai

**Affiliations:** National Heart, Lung, and Blood Institute, National Institutes of Health, Bethesda, Maryland.

**Keywords:** computer-aided diagnosis, image processing, magnetic resonance imaging, myocardial blood flow, myocardial perfusion, quantification

## Abstract

**OBJECTIVES:**

The authors developed a fully automated framework to quantify myocardial blood flow (MBF) from contrast-enhanced cardiac magnetic resonance (CMR) perfusion imaging and evaluated its diagnostic performance in patients.

**BACKGROUND:**

Fully quantitative CMR perfusion pixel maps were previously validated with microsphere MBF measurements and showed potential in clinical applications, but the methods required laborious manual processes and were excessively time-consuming.

**METHODS:**

CMR perfusion imaging was performed on 80 patients with known or suspected coronary artery disease (CAD) and 17 healthy volunteers. Significant CAD was defined by quantitative coronary angiography (QCA) as ≥70% stenosis. Nonsignificant CAD was defined by: 1) QCA as <70% stenosis; or 2) coronary computed tomography angiography as <30% stenosis and a calcium score of 0 in all vessels. Automatically generated MBF maps were compared with manual quantification on healthy volunteers. Diagnostic performance of the automated MBF pixel maps was analyzed on patients using absolute MBF, myocardial perfusion reserve (MPR), and relative measurements of MBF and MPR.

**RESULTS:**

The correlation between automated and manual quantification was excellent (r = 0.96). Stress MBF and MPR in the ischemic zone were lower than those in the remote myocardium in patients with significant CAD (both p < 0.001). Stress MBF and MPR in the remote zone of the patients were lower than those in the normal volunteers (both p < 0.001). All quantitative metrics had good area under the curve (0.864 to 0.926), sensitivity (82.9% to 91.4%), and specificity (75.6% to 91.1%) on per-patient analysis. On a per-vessel analysis of the quantitative metrics, area under the curve (0.837 to 0.864), sensitivity (75.0% to 82.7%), and specificity (71.8% to 80.9%) were good.

**CONCLUSIONS:**

Fully quantitative CMR MBF pixel maps can be generated automatically, and the results agree well with manual quantification. These methods can discriminate regional perfusion variations and have high diagnostic performance for detecting significant CAD. (Technical Development of Cardiovascular Magnetic Resonance Imaging; NCT00027170)

Cardiac magnetic resonance (CMR) perfusion imaging has good diagnostic accuracy for detecting significant coronary artery disease (CAD) (1–3). Quantitative evaluation of dynamic contrast enhancement from the CMR perfusion time-signal intensity curves also accurately assess the severity of stenosis and myocardial ischemia in patients with known or suspected CAD (4–8).

There is an increased interest in fully quantitative assessment of myocardial blood flow (MBF) from CMR because it provides a wider range of perfusion estimates than semiquantitative perfusion indexes (9,10). It outperforms semiquantitative measures of perfusion and qualitative approaches in diagnosing patients with significant CAD (7,8). Absolute MBF estimates from CMR have been validated against microspheres (10,11) and positron emission tomography (PET) measurements of MBF (12–14). Preliminary studies that quantified MBF at a pixel level also validated these methods with microspheres (15), phantoms (16), and PET measurements (17). However, these validation studies required manual processing to delineate myocardial regions of interest (ROIs) and to quantify MBF. Those manual steps inevitably introduced interobserver and intraobserver variability and created large barriers that prevented routine clinical usage.

In this study, we presented a fully automated image processing framework for quantitative pixel-wise assessment of MBF using first-pass CMR perfusion imaging. This work addressed the limitations of our previous techniques to generate perfusion pixel maps (15), which required manual image segmentation and were described as laborious and time-consuming (18,19). Most subcomponents of this automated framework were previously validated (15,20–22).

To evaluate the performance of automatically generated MBF pixel maps from the proposed framework, we aimed to: 1) compare fully automated and manual measurements of MBF; 2) characterize MBF and myocardial perfusion reserve (MPR) in healthy subjects and in patients; and 3) determine the diagnostic accuracy of absolute and relative measurements of MBF and MPR in patients with known or suspected CAD.

## METHODS

### STUDY POPULATION.

Ninety-seven subjects were evaluated in this study, including 80 patients with known or suspected CAD and 17 healthy volunteers. This was a retrospective study of CMR stress and rest perfusion scans acquired as part of a clinical research protocol approved by the institutional review board of the National Heart, Lung, and Blood Institute. All subjects gave written informed consent (NCT00027170). Healthy volunteers were recruited specifically for validation purposes and needed to have a Framing-ham risk score of <1% and no history of cardiovascular disease. Patient studies were selected from the same time period as the healthy volunteers based on availability of invasive coronary angiography or coronary computed tomography angiography (CTA) within 90 days of the CMR scan. Patients were excluded if there was a change in symptoms in cases in which coronary angiography preceded CMR, if there revascularization occurred between the 2 studies, or if the digital angiography was not available for quantitative coronary angiography (QCA). Patients with CTA were excluded if they had an Agatston calcium score >0 or >30% noncalcified plaque in any major vessel.

### DEFINITION OF SIGNIFICANT CAD.

Significant CAD was defined as ≥70% stenosis in at least 1 major vessel or >50% stenosis in the left main coronary artery as confirmed by QCA. Nonsignificant CAD was defined by: 1) QCA of <70% stenosis; or by 2) CTA with <30% stenosis and a calcium score of 0 in all major vessels. QCA was performed by a physician blinded to the CMR perfusion results (Syngo QCA, Siemens Healthcare, Erlangen, Germany). CTA studies were performed on a 320-detector row scanner (Aquilion ONE, Toshiba, Otawara, Japan) and interpreted independently of CMR.

### CMR PERFUSION IMAGING.

CMR perfusion imaging was performed on a 1.5-T scanner (Siemens Healthcare, Erlangen, Germany). All subjects were instructed to abstain from caffeinated products for at least 24 h before the scan. Stress perfusion imaging was performed 70 s after a 400-mg intravenous bolus of regadenoson. Aminophylline 100 to 150 mg intravenous slow infusion was administered after stress imaging to minimize the residual effects of vasodilation. Perfusion at rest was performed 20 min later. A dose of 0.05 mmol/kg gadolinium intravenous at 2 to 5 ml/s (diethylenetriamine-pentaacetate, Magnevist, Berlex Laboratories, Wayne, New Jersey) was used and flushed with saline at 5 ml/s.

The perfusion imaging involved a steady-state free precession dual-sequence technique (23). The dual-sequence method acquired a low-resolution arterial input function (AIF) image, and 3 myocardial images every RR interval for 60 heart beats. Typical imaging parameters for the myocardial series included a nonslice selective 90° composite saturation preparation pulse, 90-ms inversion time, 1.2-ms echo time, 2.3-ms repetition time, 50° flip angle, 8-mm slice thickness, 360- × 270-mm field of view, 128 × 80 acquisition matrix, 256 × 192 image matrix, and a parallel imaging factor of 2 (24). For the AIF series, a fast low-angle shot sequence was used with a separate saturation pulse, 8° flip angle, 5.0-ms inversion time, 0.7-ms echo time, 1.3-ms repetitive time, 10-mm slice thickness, and 64 × 48 acquisition and image matrix size. Two proton density weighted images were also acquired for correcting surface-coil related intensity inhomogeneity.

### CMR IMAGE PROCESSING.

**FIGURE 1:**
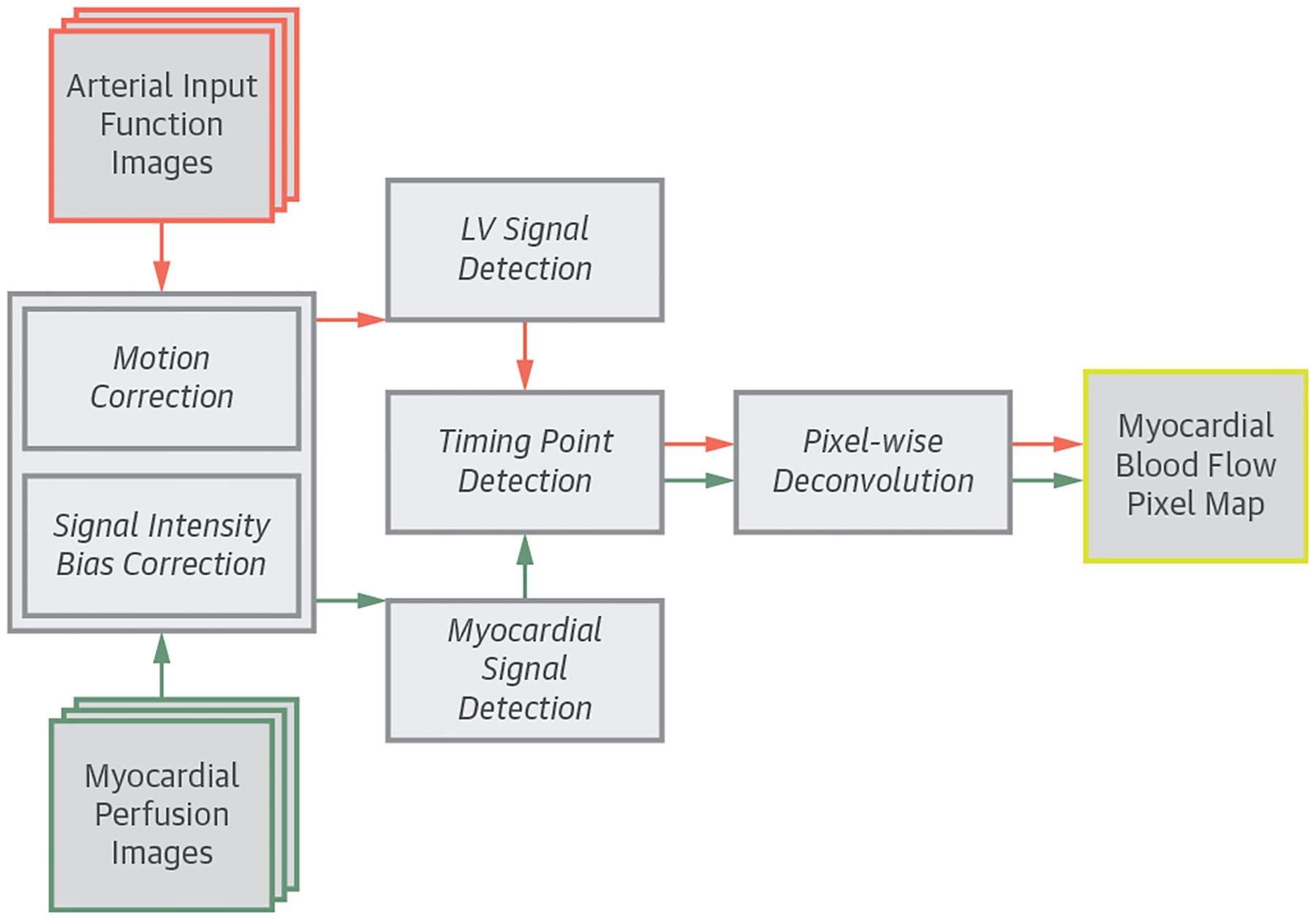
Flowchart of the Automated Pixel-Wise MBF Quantification Pipeline The automated processing pipeline for first-pass cardiac magnetic resonance myocardial blood flow (MBF) map quantification. LV = left ventricular.

The schematic diagram of the fully automated CMR perfusion quantification pipeline is shown in Figure 1. The system performs the following computer vision and image processing techniques on raw Digital Imaging and Communications in Medicine images to generate fully quantitative MBF pixel maps without any operator interaction. First, it corrects heart motion and corrects for surface coil intensity variations (20,22). It then detects the AIF and myocardial ROIs to extract time-signal intensity curves (21). Next, key time points during first-pass contrast enhancement are detected. Finally, the system deconvolves the AIF and myocardial time-signal intensity curves on a pixel-by-pixel basis to obtain MBF estimates and generates fully quantitative MBF maps (15). Detailed technical descriptions and discussion of the automated processing framework is provided in the Online Appendix.

The automated framework was implemented in Interactive Data Language (Harris Corporation, Boulder, Colorado), Java programming language Digital Imaging and Communications in Medicine interface (DCM4CHE [25]), and C/C++ programming language with the Intel Math Kernel Library (Santa Clara, California) under the Microsoft operating system (Redmond, Washington). To improve computation speed, several components use multi-threaded programming to take advantage of the multicore architecture in the CPU. Execution time to process MBF pixel maps was measured on a desktop computer with an Intel Core i7-6950X 3.0-GHz CPU using a 64-bit Microsoft Windows 10 operation system.

### COMPARISON OF AUTOMATED VERSUS MANUAL QUANTIFICATION.

Manual processing of the MBF quantification was performed on the 17 healthy subjects based on the pixel-wise perfusion analysis workflow described previously (15). Briefly, endocardial and epicardial borders of the left ventricular (LV) myocardium were manually traced on the motion-corrected images to define the myocardial ROIs and to extract pixel-wise, time-signal intensity curves. An additional ROI was drawn in the blood pool of the low-resolution AIF images to compute the LV time-signal intensity curve. Next, contrast enhancement timing points were manually identified from the LV and myocardial time-signal intensity curves to facilitate pixel-wise MBF quantification.

The automated MBF pixel maps were then compared with the manually quantified MBF pixel maps based on an 18-segment model. This was performed by manually tracing endocardial and epicardial borders on the automatically generated MBF maps. The myocardial region was then divided into 6 transmural sectors for all 3 slices, which resulted in a total of 612 segments for the comparison.

### COMPARISON OF NORMAL VERSUS ABNORMAL PERFUSION.

To compare perfusion in patients versus healthy volunteers, stress MBF, rest MBF, and myocardial perfusion reserve (MPR) were analyzed in 6 sectors per slice on the automated perfusion maps of the patients. MPR was calculated as stress MBF divided by rest MBF. In patients with significant CAD, MBF and MPR in ischemic sectors were compared with the remote sectors. Perfusion measurements from the remote zone of the patients were also compared with comparable measurements from the healthy volunteers.

### DIAGNOSTIC ACCURACY EVALUATION.

CMR measurements of MBF and MPR were evaluated on an 18-segment model by dividing the automated perfusion map of each slice into 6 transmural sectors and then averaging 2 adjacent sectors in each slice, for a total of 9 coronary territories per-patient mapping to 3 coronary arteries (Table 1). No adjudication was performed on the assignment of myocardial segments to different coronary territories to avoid subjective elements.

Relative MBF (rMBF) measurement was computed as the ratio of stress MBF in each coronary territory to a remote territory showing the most normal hyperemic blood flow. Relative MPR (rMPR) measurement was computed in a similar way. The minimum values of each measurement were evaluated by receiver-operating characteristic (ROC) curve analysis for detecting significant CAD on a per-patient and on a per-vessel basis.

### STATISTICAL ANALYSIS.

Statistics were calculated with SPSS version 19 (IBM, Armonk, New York). Continuous variables are expressed as mean ± SD for normally distributed data or median with inter-quartile range for non-normally distributed variables. Automatically and manually estimated MBF were compared with Pearson’s correlation and Bland-Altman plots. A Mann-Whitney test was used to compare MBF and MPR estimates between groups. A p value <0.05 was considered statistically significant. Diagnostic accuracy analysis was performed using MedCalc statistical software (Ostend, Belgium) with a DeLong test to compare the differences in areas under the curve (AUC) in ROC analysis. Optimal threshold values were determined with the Youden index and the balance between sensitivity and specificity. A McNemar test was used to compare the sensitivity and specificity among different perfusion measurements.

## RESULTS

Ninety-seven subjects, including patients with known or suspected CAD (n = 80) and healthy volunteers (n = 17), were enrolled. Table 2 shows the clinical characteristics of the patients and healthy volunteers. Patients with known or suspected CAD were generally older, included smokers, and had more cardiovascular risk factors than healthy volunteers. The patient groups were on a wide range of medications. Similar cholesterol levels between groups might be due to statin treatment.

Invasive coronary angiography was performed in 48 patients with a median of 13 days between CMR and angiography (95% confidence interval: 6 to 20). CTA was performed in 32 patients, and 24 of them had CMR on the same day. The prevalence of significant CAD was 44% (35 of 80 patients). Among the patients with significant CAD, 63% (n = 22) had single-vessel disease and 37% (n = 13) had multivessel disease, including 4 patients with 3-vessel or left main CAD as assessed by QCA. On a per-vessel level, 22% (52 of 240 vessels) of the coronary arteries had significant stenosis, which included 25 left anterior descending (LAD), 10 circumflex (CX), and 17 right (RCA) coronary arteries.

CMR images for the entire study were processed automatically with identical settings and parameters in a batched script. No studies were excluded or failed in the processing. Computational time averaged 46.9 ± 4.3 s/slice.

**FIGURE 2:**
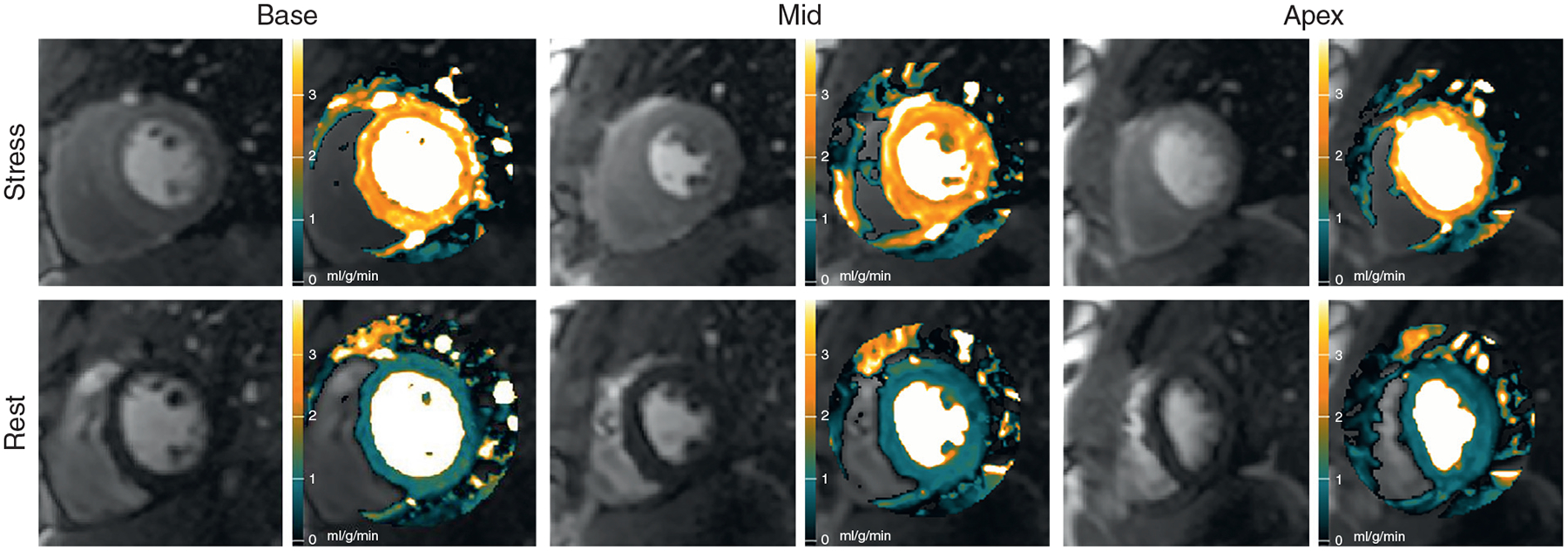
Automated MBF Pixel Maps in a Normal Volunteer Automated stress and rest myocardial blood flow (MBF) pixel maps in a normal volunteer show coherent hyperemic MBF **(orange)** and rest MBF **(green)** on all 3 slices (Online Video 1).

Automated MBF maps from a healthy volunteer (Figure 2, Online Video 1) showed a coherent stress MBF at approximately 3.15 ml/g/min (orange color), whereas the rest perfusion maps showed a uniform MBF at approximately 1.08 ml/g/min (green color) on all 3 slices.

**FIGURE 3:**
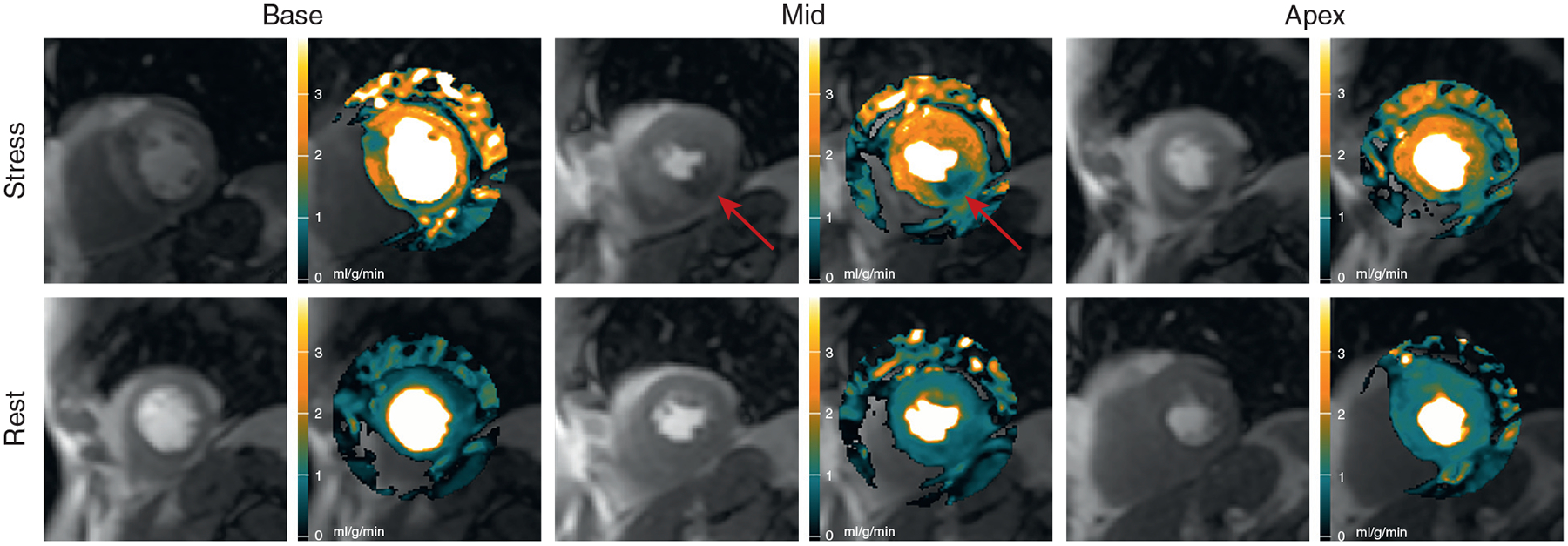
Automated MBF Pixel Maps in Patient With Single-Vessel Disease Automated MBF pixel maps in a patient with 89% right coronary artery stenosis by QCA show an inferior perfusion defect **(red arrows)** on the stress perfusion image and MBF map (Online Video 2). The possible perfusion defect in the basal anteroseptal wall did not reach abnormal thresholds. It was associated with a severe narrowing of a septal perforator artery that was too small for QCA. MBF = myocardial blood flow; QCA = quantitative coronary angiography.

Automated MBF maps from a patient with single-vessel disease are shown in Figure 3 and Online Video 2. This patient had an 89% RCA stenosis confirmed by QCA. The stress maps showed an inferior perfusion defect in the mid-ventricular and apical slices. Stress MBF on the mid-ventricular slice fell within the green color range but had subendocardial regions that fell in the black color range. Thus, stress MBF in the inferior wall (0.77 ml/g/min) was comparable or lower than rest perfusion (0.81 ml/g/min), whereas remote myocardium was in the hyperemic range (2.51 ml/g/min).

**FIGURE 4:**
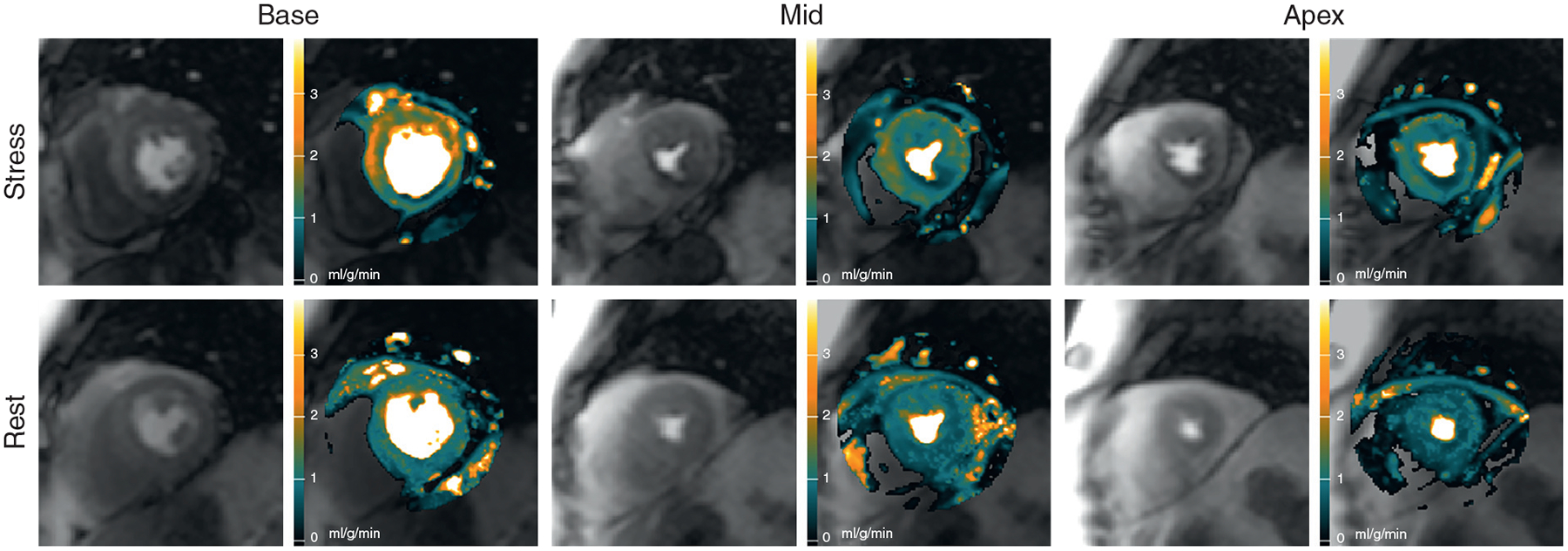
Automated MBF Pixel Maps in Patient With Multivessel Disease Automated MBF pixel maps in a patient with an 87% circumflex stenosis, an 84% left anterior descending stenosis, and a 65% right coronary artery stenosis by QCA show corresponding perfusion defects in the stress maps in all 3 coronary artery territories. There is some epicardial hyperemic perfusion in the basal anteroseptal and anterolateral segments (Online Video 3). MBF = myocardial blood flow; QCA = quantitative coronary angiography.

A patient with multivessel disease is shown in Figure 4 and Online Video 3. This patient had an 87% stenosis in the CX, an 84% stenosis in the LAD, and a 65% stenosis in the RCA by QCA. The stress maps showed perfusion defects in all 3 coronary artery territories, but the basal slice showed a modest hyperemic epicardial response in the anteroseptal and anterolateral segments.

### COMPARISONS OF AUTOMATICALLY VERSUS MANUALLY QUANTIFIED MBF.

**FIGURE 5:**
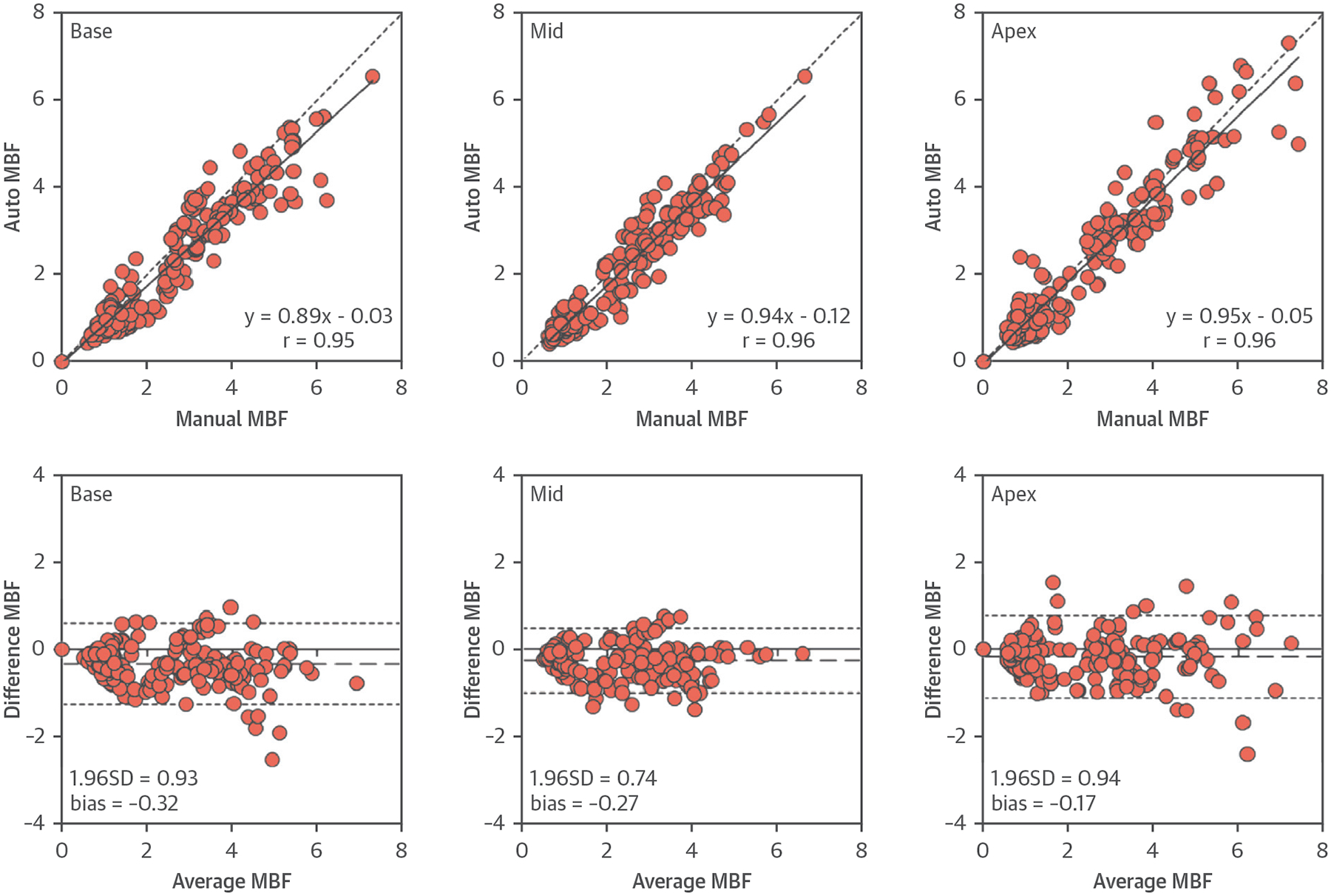
Comparisons of MBF Between Automated and Manual Quantification Correlations and Bland-Altman plots comparing automatically and manually quantified MBF in healthy volunteers. The **dashed lines** represent the bias (MBF_automated_ − MBF_manual_) and limits of agreement (mean ± 1.96 SD). MBF = myocardial blood flow.

There was an excellent correlation between the automated and manual MBF in all slices (r = 0.96) and slice-by-slice comparisons (Figure 5) (r = 0.95, 0.96, and 0.96 for base, mid, and apex, respectively). Bland-Altman analysis showed that automatic measurements slightly underestimated MBF compared with the manual measurements in all slices (bias = −0.25 ml/g/min; p < 0.001) and individual slice comparisons (bias = −0.32, −0.27, and −0.17 ml/g/min for base: p = 0.01, mid: p = 0.01, and apex: p = NS, respectively).

### COMPARISONS OF MBF AND MPR IN PATIENTS VERSUS THE HEALTHY VOLUNTEERS.

**FIGURE 6:**
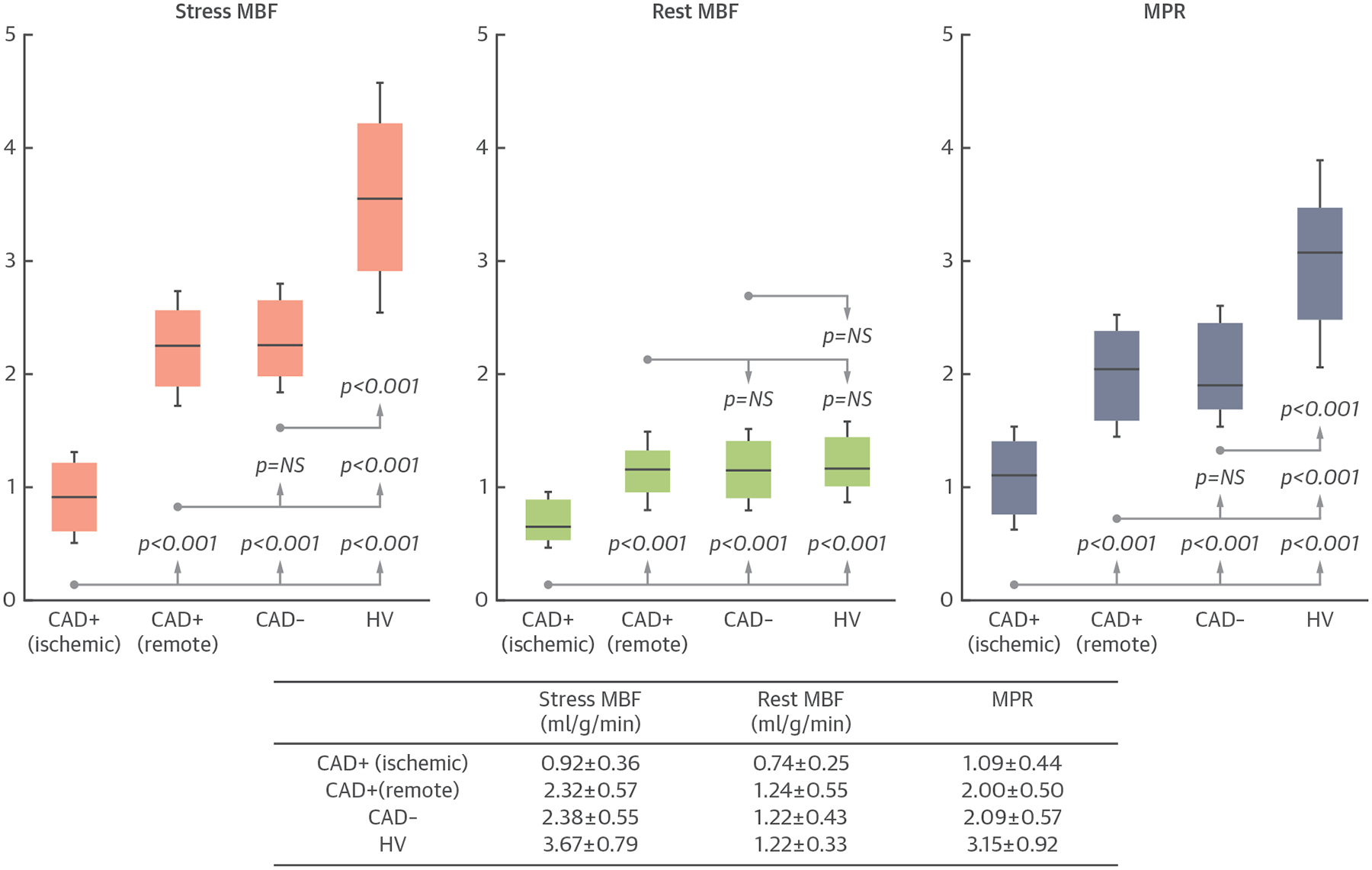
Comparisons of MBF Between Patients and Healthy Volunteers In patients with significant coronary artery disease (CAD+), myocardial blood flow (MBF) and myocardial perfusion reserve (MPR) in the ischemic sectors are significantly lower than in the remote sectors (all p < 0.001). However, MBF and MPR in the remote sectors of CAD+ patients are not significantly different than patients without significant CAD (CAD−) (all p = NS). Stress MBF and MPR in CAD− patients and the remote sector of CAD+ patients are lower than the healthy volunteers (both p < 0.001). HV = healthy volunteers.

In patients with significant CAD, perfusion in the ischemic sector was significantly lower than the remote sectors for stress MBF, rest MBF, and MPR (Figure 6) (all p < 0.001). Remote sector stress MBF, rest MBF, and MPR were not different in patients with significant CAD versus patients without CAD (all p = NS). However, stress MBF and MPR in the remote zone of the 2 patient groups were significantly lower than in the healthy volunteers (all p < 0.001), whereas the rest MBF in the remote zone was similar (both p = NS).

### DIAGNOSTIC PERFORMANCE.

**FIGURE 7:**
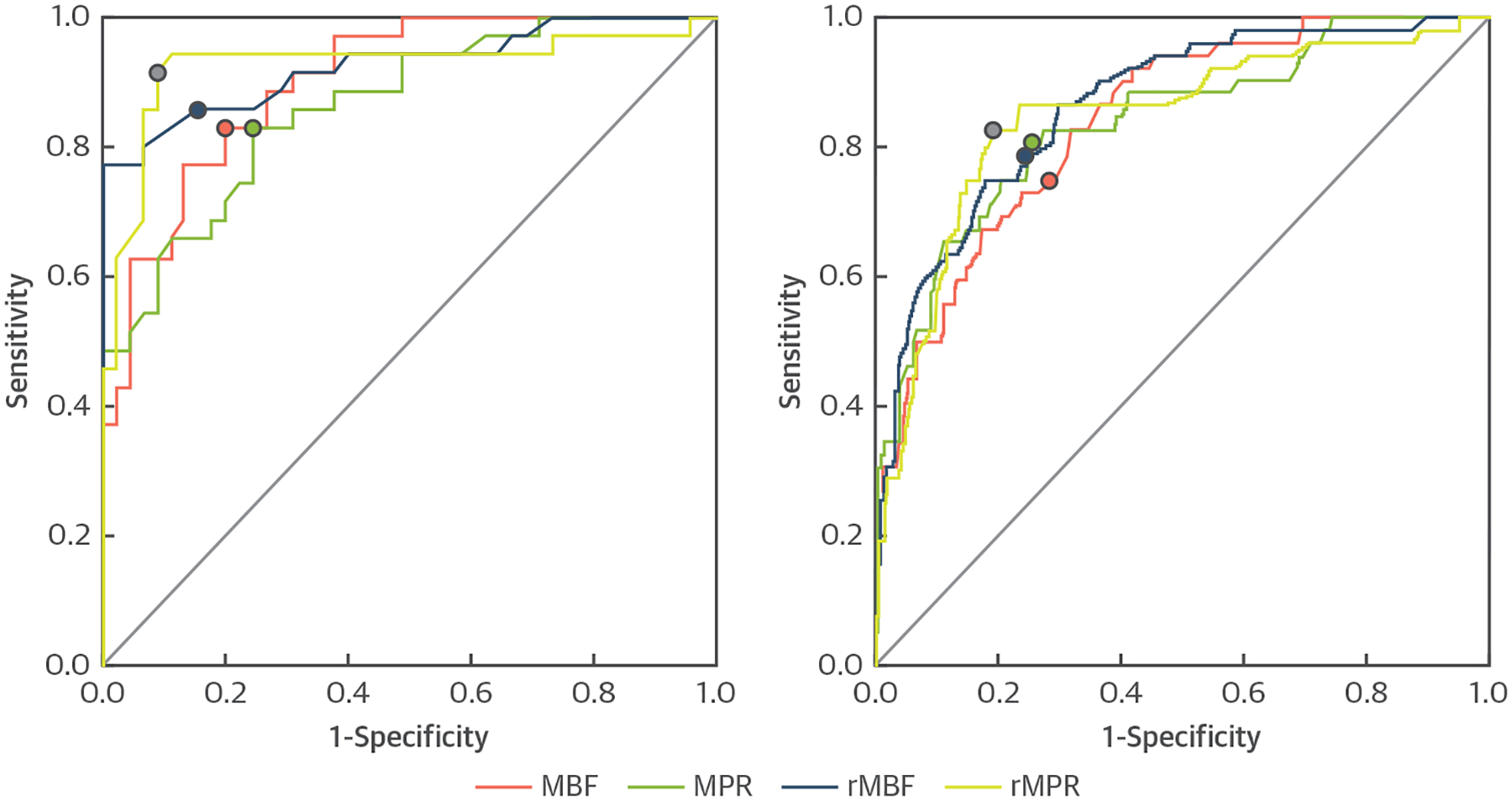
Diagnostic Accuracy Comparisons Receiver-operating characteristic curves show the diagnostic performance of fully automated CMR perfusion quantification by myocardial blood flow (MBF), myocardial perfusion reserve (MPR), relative MBF (rMBF), and relative MPR (rMPR) on a per-patient and per-vessel analysis.

On a per-patient basis, both MBF and MPR had excellent AUCs of 0.901 and 0.864, respectively (Figure 7, Table 3). Using a threshold of 1.290 for stress MBF and a threshold of 1.475 for MPR, both had similar sensitivity, specificity, and accuracy. For relative perfusion analysis, both rMBF and rMPR had excellent AUCs of 0.925 and 0.926, respectively. Using a threshold of 0.575 for rMBF and a cutoff of 0.770 for rMPR, the sensitivity, specificity, and accuracy were very good.

On a per-vessel basis, the ROC curves for all 4 quantitative indexes were similar (Figure 7, Table 3). The AUC ranged from 0.837 to 0.864 (Figure 7, Table 3). The optimal thresholds were 1.350 for stress MBF, 1.435 for MPR, 0.605 for rMBF, and 0.775 for rMPR. There were no significant differences in AUCs, sensitivities, specificities, and accuracies among the different perfusion metrics on per-patient− and on per-vessel based− analyses (Figure 7, Table 3) (all p = NS).

## DISCUSSION

We developed a fully automated system for quantifying MBF pixel maps from contrast-enhanced first-pass CMR perfusion images in near real-time and showed its clinical diagnostic performance to detect significant CAD in patients. Our system removed the need for time-consuming manual processes in CMR perfusion quantification. It also alleviated the interobserver and intraobserver variability issues that frequently occurred during manual perfusion quantification. Stress MBF and MPR, as well as regional relative rMBF and rMPR measured from the automated pixel maps, all performed well for detecting significant CAD. This study was an important step toward the overall feasibility, performance, and validation of fully automated CMR perfusion quantification in patients with CAD.

The individual components of the automated processing system used in this study were extensively validated, including the motion-correction pipeline (20), the surface coil intensity correction (22), the AIF and timing point detection (21), and the pixel-wise MBF quantification (15) (Online Appendix). The present study integrated these step-by-step processes into a unified package. Although not demonstrated in this study, the described methods have also been applied to various imaging sequences (20,21,26,27) and different imaging protocols, such as using a dual-bolus AIF acquisition (8,9,15). Furthermore, the system was vendor and platform independent as opposed to automatic inline processing that requires dedicated hardware and pulse sequence implementations (28). Our universal framework could streamline processing of large-scale data sets on generic desktop computers. For example, it could batch process all perfusion images in this study with identical settings.

From a cardiology perspective, tremendous effort has been placed in developing various quantitative myocardial perfusion imaging modalities. This goal is becoming feasible for CMR. Our MBF quantification approach followed the simple indicator dilution principles described by Zierler (28,29). In that era, measuring absolute concentrations of indicators was not feasible, but Zierler noted that blood flow in units of milliliters per gram per minute could be derived accurately based on mean transit time and did not require absolute indicator concentration quantification.

Our MBF and MPR estimates from healthy volunteers were similar to other recent CMR studies that used Fermi modeling methods. Broadbent et al. (30) reported rest perfusion values of 1.5 ± 0.5 ml/g/min and Kellman et al. (31) measured rest perfusion at 0.95 ml/g/min from normal volunteers. Our stress MBF estimates were also comparable to the values reported by Broadbent et al. (30) at 3.8 ± 1.0 ml/g/min and Kellman et al. (31) at 3.4 ml/g/min. For perfusion in patients, our MBF and MPR estimates were commensurate with the values summarized by Biglands et al. (32) in patients with CAD, whereas our perfusion estimates were lower than their values in patients without CAD.

The MBF and MPR measurements in our study also corresponded well with expected values in normal subjects based on PET perfusion publications (33). Normal PET stress perfusion averaged 3.16 ± 0.85 ml/g/min (range 1.86 to 5.05 ml/g/min) in various publications, and rest perfusion averaged 0.84 ± 0.16 ml/g/min (range 0.61 to 1.24 ml/g/min). MPR averaged 4.11 ± 1.23 (range 3.16 to 4.99). Compared with PET perfusion, rest MBF estimates by CMR were on the higher side. Different implementations of quantification methods and imaging protocols could be important factors that contribute to different ranges of MBF estimation.

In our study, patients in whom significant CAD was excluded had lower stress MBF and MPR than normal healthy volunteers. Sdringola et al. (33) noted reduced stress MBF in purpose-recruited asymptomatic volunteers who had undisclosed risk factors and nicotine in their blood streams. The obvious differences in stress perfusion between healthy volunteers and patients without epicardial CAD might be part of the reason why relative measurements of rMBF and rMPR trend toward better diagnostic accuracy than absolute measurements of MBF and MPR. Because CAD affects perfusion in an intrinsic region-by-region basis, regionally heterogeneous perfusion related to the branching pattern of the coronary artery is fundamental for detecting CAD. Fractional flow reserve also relies on relative measurement of pressure differences.

Detection of truly balanced ischemia is one presentation of CAD that could benefit from quantification of absolute MBF or MPR. In our data, the regional rMBF and rMPR correctly detected all patients with 3-vessel and left main disease, but the sample size (n = 4) was too small for this type of patient to make a broader statement about the performance of this automated method in detecting balanced ischemia. Small vessel disease in patients with suspected CAD or normal epicardial coronary arteries is another situation in which quantification might have benefits. The latter conditions will need to be discriminated from inadequate responses to vasodilation, a problem that some groups are addressing with the splenic response to adenosine (34). Unfortunately, that method does not seem to apply to regadenoson.

All 4 of our quantitative metrics provided per-patient diagnostic accuracy results that were equivalent or more favorable than the pooled diagnostic performance summarized in a meta-analysis of quantitative CMR perfusion methods by van Dijk et al. (35). They reported a sensitivity of 83% (range 75% to 88%), specificity of 76% (range 65% to 85%), and AUC of 0.87 (range 0.84 to 0.90). On a per-vessel basis, 3 of 4 quantitative metrics fall within the diagnostic accuracy ranges summarized in that meta-analysis.

### STUDY LIMITATIONS.

Our reference standard by invasive QCA and CTA might not reflect microvascular disease in patients. Intracoronary pressure-derived fractional flow reserve measurements might more accurately assess the physiological significance of coronary artery stenosis. QCA is not able to assess blood flow supply through collateral vessels. Further studies are required to compare this with absolute MBF measured from independent reference standards.

Although the automatic MBF measurements agreed well with manual quantification, one should not over-interpret the differences in the Bland-Altman plots. Inclusion of some blood cavity pixels can bias manual measurements and could account for some of the differences observed.

The optimum thresholds selected from different perfusion values were tested on the same data as the accuracy evaluation. Until verified in independent datasets, the thresholds should be considered conceptually important but not necessarily generalizable.

## CONCLUSIONS

Our fully automated system alleviated the laborious manual processes for CMR perfusion quantification. The automated MBF maps had high diagnostic performance for detecting significant CAD in patients.

## Supplementary Material

MMC2

MMC3

MMC4

MMC1

## Figures and Tables

**TABLE 1:** Assignment of 18 Myocardial Segments to LAD, RCA, and CX Coronary Artery Territories at the Basal, Mid, and Apical Left Ventricle

	LAD	RCA	CX
Basal	Anterior	Inferior	Anterolateral
	Anteroseptal	Inferoseptal	Inferolateral
Mid	Anterior	Inferior	Anterolateral
	Anteroseptal	Inferoseptal	Inferolateral
Apical	Anterior	Inferior	Anterolateral
	Anteroseptal	Inferoseptal	Inferolateral

CX = circum flex; LAD = left anterior descending; RCA = right coronary artery.

**TABLE 2:** Patient Demographics and Clinical Characteristics

	CAD+Patients (n = 35)	CAD-Patients (n = 45)	Healthy Volunteers (n = 17)
Risk factors for CAD			
Male	21 (60.0)	21 (46.7)	15 (88.2)
Age, yrs	62.3 ± 11.0	53.8 ± 13.3	23.0 ± 7.3
Body mass index, kg/m^2^	27.8 ± 5.1	28.3 ± 7.5	25.1 ± 3.0
Current smokers	6 (17.1)	3 (6.7)	0 (0.0)
Hypertension	30 (85.7)	25 (55.6)	0
Diabetes	8 (22.9)	8 (17.8)	0
Hyperlipidemia	31 (88.6)	25 (55.6)	0
Total cholesterol, mg/dl	156.5 ± 36.7	180.4 ± 38.8	146.5 ± 33.4
LDL cholesterol, mg/dl	77.8 ± 33.2	100.0 ± 33.3	85.9 ± 30.6
HDL cholesterol, mg/dl	45.3 ± 14.8	54.5 ± 19.5	47.7 ± 15.5
Triglyceride, mg/dl	129.6 ± 54.0	102.2 ± 54.3	64.8 ± 29.9
Medications	33 (94.3)	34 (75.6)	0
Statin	28 (80.0)	20 (44.4)	0
Beta-blocker	21 (60.0)	12 (26.7)	0
Aspirin	26 (74.3)	17 (37.8)	0
ACE inhibitor	18 (51.4)	12 (26.7)	0
Calcium channel blocker	5 (14.3)	5 (11.1)	0
Nitrate	7 (20.0)	3 (6.7)	0
CAD status			
Previous PCI	13 (37.1)	4 (8.9)	0
Current CAD (QCA ≥70%)	35 (100)	0	0
1 Vessel	22 (62.9)	0	0
2 Vessels	9 (25.7)	0	0
3 Vessels	4 (11.4)	0	0

Values are n (%) or mean ± SD.

ACE = angiotensin-converting enzyme; CAD = coronary artery disease; HDL = high-density lipoprotein; LDL = low-density lipoprotein; PCI = percutaneous coronary interventions; QCA = quantitative coronary angiography.

**TABLE 3:** Diagnostic Performance Comparisons of CMR Perfusion Quantification by MBF, MPR, rMBF, and rMPR From Automated Perfusion Maps

	AUC	95% CI	Threshold	Sensitivity (%)	Specificity (%)	Accuracy (%)
Per-patient diagnostic performance						
MBF	0.901	0.837–0.964	1.290	82.9	80.0	81.3
MPR	0.864	0.785–0.942	1.475	82.9	75.6	78.8
rMBF	0.925	0.863–0.988	0.570	85.7	84.4	85.0
rMPR	0.926	0.856–0.997	0.770	91.4	91.1	91.3
Per-vessel diagnostic performance						
MBF	0.841	0.784–0.898	1.350	75.0	71.8	72.5
MPR	0.837	0.773–0.902	1.435	80.8	74.5	75.8
rMBF	0.864	0.809–0.919	0.605	78.8	75.5	76.3
rMPR	0.844	0.778–0.909	0.775	82.7	80.9	81.3

Diagnostic performance comparisons of CMR perfusion quantification by myocardial blood flow (MBF), myocardial perfusion reserve (MPR), relative MBF (rMBF), and relative MPR (rMPR) from automated perfusion maps (see Figure 7 for receiver-operating curves).

AUC = area under the curve; CI = confidence interval.

## PERSPECTIVES

### COMPETENCY IN MEDICAL KNOWLEDGE:

First-pass CMR perfusion imaging can accurately assess the severity of stenosis and myocardial ischemia in patients with known or suspected CAD. Quantitative CMR perfusion provides a unique tool for objective evaluation of global and regional blood flow in the myocardium to assist the diagnosis of ischemic and nonischemic cardiomyopathy.

### TRANSLATIONAL OUTLOOK:

This study markedly improves the workflow of CMR perfusion quantification before clinical adaptation. We developed a fully automated technique that can objectively quantify CMR perfusion images and generate MBF pixel maps in near real-time. We showed the automated MBF maps are comparable to manual quantification and have high diagnostic accuracy to detect CAD. Future trials are needed to evaluate the efficacy of fully automated MBF pixel maps for clinical applications.

## ABBREVIATIONS AND ACRONYMS

AIF: arterial input function
AUC: area under the curve
CAD: coronary artery disease
CMR: cardiac magnetic resonance
CTA: computed tomography angiography
CX: circumflex coronary artery
LAD: left anterior descending artery
LV: left ventricular
MBF: myocardial blood flow
MPR: myocardial perfusion reserve
PET: positron emission tomography
QCA: quantitative coronary angiography
rMBF: relative myocardial blood flow
rMPR: relative myocardial perfusion reserve
RCA: right coronary artery
ROC: receiver-operating characteristic
ROI: region of interest
