# Supplementary material for: Diagnostic Performance of Fully Automated Pixel-Wise Quantitative Myocardial Perfusion Imaging by Cardiovascular Magnetic Resonance
Source: JACC Cardiovasc Imaging. Author manuscript; Available in PMC 2022 Jan 15. (PMC8760891; doi:10.1016/j.jcmg.2018.01.005)
Supplement: MMC1 [file NIHMS968296-supplement-MMC1.docx]

**Appendix**

As shown in Figure-1, the fully automated CMR perfusion quantification system performs a series of image processing steps starting with raw DICOM images and finishing with fully quantitative MBF pixel maps.

***Motion Correction***

All raw perfusion image series were first automatically processed by a non-rigid motion correction technique developed in our previous work (1). This method combines discrete feature matching for large displacement estimation with a dense variational optical flow formulation in a multithreaded architecture. The system then uses post-hoc interpolation warping following the nonrigid displacement estimation to correct motions and preserved perfusion dynamics.

This automated method handles CMR perfusion series acquired with different pulse sequences during a breath-hold and during free-breathing to minimize frame-to-frame cardiac motion. The method can also compensate for motion between myocardial perfusion images and auxiliary images such as the AIF images and PD-weighted images which are acquired with markedly different resolution, appearance, and imaging methods. Importantly, this approach was shown to preserve the signal intensity dynamics crucial for qualitative and quantitative assessments.

***Surface-Coil Intensity Correction***

An automated method based on our previous work (2,3) was incorporated to correct B_1_-field related inhomogeneity in the perfusion images. This method classifies multiple regions of interest from the motion corrected PD images to group tissues with similar signal intensity. A third-order polynomial surface fit was then used to approximate the signal intensity bias field based on a hierarchical weighting scheme applied to different regions. The estimated intensity bias field was subsequently applied to the motion corrected T1 perfusion images to correct surface-coil related intensity inhomogeneity. This method has been shown to improve the homogeneity of myocardial signal intensity as well as lower the spatial variation of MBF on normal myocardium (3,4).

***Time-Signal Intensity Curve Detection***

We have previously developed an automated method to measure the AIF based on time-signal intensity changes of the LV cavity in the CMR perfusion images (5). This method uses independent component analysis technique to separate the left and the right ventricles from the perfusion images and to extract the AIF signal from the LV. This method effectively selects bright LV pixels, excludes papillary muscles, and removes the subjectivity of manual measurement of the AIF. Automatically measured AIFs were in good agreement with manual measurements and produced similar MBF estimates (5).

An extension of this automated method is also applied to detect the LV myocardial region to obtain myocardial time-signal intensity curves. This extended method includes feature classification based on multi-parametric maps of correlation, peak time, and peak value calculated from the perfusion series. Region growing, active contour, and morphological image processing steps are incorporated to constrain myocardial region detection. Finally, a reference region is extracted from the myocardial mask to calculate a reference myocardial intensity curve.

***Contrast Enhancement Timing Point Detection***

From the auto-detected AIF and reference myocardial time-signal intensity curves, important time-signal features such as contrast enhancement start and peak time are automatically detected. This step is based on our previous method using a line fit to the contrast enhancement upslope to obtain the contrast start time (5). The baseline intensity range of the perfusion series can also be detected by this method.

This automated method agreed well with manual analysis to detect the start time of the contrast enhancement. This timing point is used to extract the first-pass contrast dynamic range from both AIF and myocardial intensity curves for perfusion quantification. This automated timing point detection can also facilitate temporal alignment of AIF and myocardial image series obtained from separated acquisitions such as using a dual-bolus AIF imaging protocol (6).

***Pixel-Wise Deconvolution***

From the central volume principle described by Zierler for indicator-dilution experiments, the signal intensity observed in the myocardium is represented as a convolution of the AIF and an impulse response function (7). The impulse response function can be derived through a reverse process of deconvolution (8). When the impulse response function is normalized, it becomes a probability density function that characterizes contrast transit times through the myocardial tissue. In the case where AIF and the myocardial perfusion images are obtained on different but linear scales, the conversion to a probability density function effectively accounts for the different scaling factors. The initial amplitude of the probability density function equals the blood flow through that region in response to an instantaneous input of contrast arrival. Note that Zieler defines ml/min/g or flow per unit weight as “relative perfusion” while cardiologists have subsequently considered this a measure of “absolute perfusion.”

In this study we used a logistic impulse response function as described in our previous work (9) to perform model-constrained deconvolution and quantify MBF. This logistic function adds a linear offset to the widely used Fermi function (10) during and after the first-pass, which accounts for the leakage of extracellular gadolinium contrast into the interstitial space and its slow clearance relative to the first-pass kinetics. Deconvolution of the AIF and myocardial time-signal intensity curves was performed using this model on a pixel-by-pixel basis to obtain MBF pixel maps.

A robust and relatively fast implementation of the Marquardt–Levenberg iterative algorithm was used for the nonlinear least-squares fitting in our model-constrained deconvolution (11). To increase the computation speed, this deconvolution step was implemented in multi-threaded programming to distribute the bulk of pixel-wise processing to different CPU cores to deconvolve multiple signal intensity curves simultaneously. To further save computation time, a circular-shaped mask surrounding the heart was generated based on the previously described automated LV detection to deconvolve only the pixels within this masked region.

Our previous study demonstrated the feasibility of obtaining pixel-level MBF maps from CMR perfusion scans but required manual processing steps. The pixel-wise perfusion quantification in that study was validated in an animal model and showed MBF estimates from CMR closely correlated with absolute microsphere measurements over a wide range of MBF values. This previous work provides the fundamental methodology and technical basis for this study.

***Color-Encoding Visualization***

After the pixel-wise deconvolution, the MBF maps were generated based on a calibrated color scale for visual interpretation. The color-encoded MBF pixel maps were displayed using a color look-up table representing high-to-low perfusion values in the following order: Bright orange to white color indicates myocardial regions with hyperemic MBF during stress perfusion. Green color indicates MBF value typically encountered at rest. Black color indicates lower MBF values than usually measured at rest.

The color-coded MBF maps improve visual discrimination of different perfusion values and expand the display dynamic range beyond grayscale levels. However, color-based visualization is more technically challenging than grayscale display as the calibration of different color ranges for visual perception is more laborious than the grayscale calibration. While it is beyond the scope of this study, diagnostic performance evaluation of different color-encoding schemes for visual assessment of the automated MBF maps may be of interest for future investigations.

**TECHNICAL DISCUSSION**

Several studies have worked on improving CMR myocardial perfusion analysis (12-14). In general, these earlier publications were based on automated or semi-automated image processing steps to evaluate semi-quantitative perfusion indices. Furthermore, most of these studies only evaluated the results based on small numbers of patients and did not address the ability to differentiate ischemic zone from remote myocardium during stress perfusion.

Torroni et al. (15) developed a near-automated technique to evaluate myocardial perfusion by CMR. The myocardial ROI was first segmented in a manually selected frame and propagated to consecutive frames using nonrigid registration. The perfusion was quantified segmentally to evaluate 42 patients with normal perfusion or suspected CAD using empirical parameters such as the peak signal amplitude and initial upslope. Beache et al. (16) developed an automated framework for semi-quantitative CMR perfusion analysis. Their method involves non-rigid image registration and segmentation of the myocardium for pixel-wise mapping of peak signal intensity, time to peak, initial upslope, and average plateau. The method was tested on synthetic images and 8 patients that underwent a myoregeneration therapy. Weng et al. (17) proposed a semi-automated framework to evaluate CMR perfusion. Cardiac motion was first corrected using model-based registration; the myocardial region was then manually selected for time-signal intensity correction and pixel-wise deconvolution. The results were compared to sector-based MBF quantification. However, their technique was evaluated only on rest perfusion images.

None of these studies presented fully automated and fully quantitative pixel-wise assessment of MBF during stress and at rest, and applied to patients with CAD that were confirmed with a clinically relevant reference standard such as QCA. Our study documents clinical feasibility of fully automated and fully quantitative pixel-wise assessment of MBF using first-pass CMR perfusion imaging in healthy volunteers and patients with CAD.

In terms of clinical workflow by our system, an MBF map can be quantified from a raw perfusion slice in less than 50 seconds on generic desktop computers. This requirement is practical for near real-time processing of MBF pixel maps immediately after CMR perfusion imaging is performed to provide feedback to the users. Fast off-line systems can reduce the time delay for inline CMR image processing which are dependent on dedicated reconstruction computers for the scanners. Furthermore, the processing time will likely improve as our multithreaded processing will scale favorably with increasingly sophisticated processor architectures and parallel computing implementations.

The results of our automated MBF pixel maps can be presented in both grayscale as well as color rendered images stored in DICOM format for archiving. While the grayscale images are useful for regional MBF comparisons, the color-coded images may be used as a form of computer-aided diagnosis tool to accompany the first-pass CMR perfusion images for visual assessment (as demonstrated in the supplementary videos). Nevertheless, there is a further need to assess the utility and accuracy of these automated MBF pixel maps as a primary diagnostic technique or as a secondary computer-aided diagnosis tool in large clinical trials.

As we have previously studied, signal intensity correction for T1 nonlinearity does not significantly change CMR perfusion quantification except with extreme sequence parameter selections (18). Similarly, T2* correction was also not shown to have a significant impact on MBF quantification (19). Thus, CMR perfusion quantification can be based on time-signal intensity curves that are approximately linear in response to different gadolinium concentrations rather than converting the signal intensity to the concentration units that requires exact knowledge of the sequence parameters as well as ideal modeling assumptions (8).

**APPENDIX REFERENCES**

1. Benovoy M, Jacobs M, Cheriet F, Dahdah N, Arai AE, Hsu LY. Robust universal nonrigid motion correction framework for first-pass cardiac MR perfusion imaging. J Magn Reson Imaging 2017.

2. Hsu LY, Aletras AH, Arai AE. Correcting surface coil intensity inhomogeneity improves quantitative analysis of cardiac magnetic resonance images Proceeding of 5th IEEE International Symposium on Biomedical Imaging (ISBI): From Nano to Macro, Paris, France, 2008:1425 - 1428

3. Miller CA, Hsu LY, Ta A, Conn H, Winkler S, Arai AE. Quantitative pixel-wise measurement of myocardial blood flow: the impact of surface coil-related field inhomogeneity and a comparison of methods for its correction. J Cardiovasc Magn Reson 2015;17:11.

4. Nielles-Vallespin S, Kellman P, Hsu LY, Arai AE. FLASH proton density imaging for improved surface coil intensity correction in quantitative and semi-quantitative SSFP perfusion cardiovascular magnetic resonance. J Cardiovasc Magn Reson 2015;17:16.

5. Jacobs M, Benovoy M, Chang LC, Arai AE, Hsu LY. Evaluation of an automated method for arterial input function detection for first-pass myocardial perfusion cardiovascular magnetic resonance. J Cardiovasc Magn Reson 2016;18:17.

6. Hsu LY, Rhoads KL, Holly JE, Kellman P, Aletras AH, Arai AE. Quantitative myocardial perfusion analysis with a dual-bolus contrast-enhanced first-pass MRI technique in humans. Journal of Magnetic Resonance Imaging 2006;23:315-322.

7. Zierler K. Indicator dilution methods for measuring blood flow, volume, and other properties of biological systems: a brief history and memoir. Ann Biomed Eng 2000;28:836-48.

8. Jerosch-Herold M. Quantification of myocardial perfusion by cardiovascular magnetic resonance. J Cardiovasc Magn Reson 2010;12:57.

9. Hsu LY, Groves DW, Aletras AH, Kellman P, Arai AE. A quantitative pixel-wise measurement of myocardial blood flow by contrast-enhanced first-pass CMR perfusion imaging: microsphere validation in dogs and feasibility study in humans. JACC Cardiovasc Imaging 2012;5:154-66.

10. Jerosch-Herold M, Wilke N, Stillman AE, Wilson RF. Magnetic resonance quantification of the myocardial perfusion reserve with a Fermi function model for constrained deconvolution. Med Phys 1998;25:73-84.

11. Markwardt CB. Non-Linear Least Squares Fitting in IDL with MPFIT. Proceedings of Astronomical Data Analysis Software and Systems XVIII, Quebec, Canada, ASP Conference Series 2009;411:251-254.

12. Breeuwer M, Quist M, Spreeuwers L, Paetsch I, Al-Saadi N, Nagel E. Towards automatic quantitative analysis of cardiac MR perfusion images. Int Congr Ser 2001;1230:922-927.

13. Xue H, Zuehlsdorff S, Kellman P et al. Unsupervised inline analysis of cardiac perfusion MRI. Medical Image Computing and Computer-Assisted Intervention (MICCAI): Lecture Notes in Computer Science, 2009:741-749.

14. Gupta V, Hendriks EA, Milles J et al. Fully Automatic Registration and Segmentation of First-Pass Myocardial Perfusion MR Image Sequences. Academic Radiology 2010;17:1375-1385.

15. Tarroni G, Corsi C, Antkowiak PF et al. Myocardial Perfusion: Near-automated Evaluation from Contrast-enhanced MR Images Obtained at Rest and during Vasodilator Stress. Radiology 2012;265:576-583.

16. Beache GM, Khalifa F, El-Baz A, Gimel'farb G. Fully automated framework for the analysis of myocardial first-pass perfusion MR images. Med Phys 2014;41.

17. Weng AM, Ritter CO, Beer M, Hahn D, Kostler H. Quantitative pixelwise myocardial perfusion maps from first-pass perfusion MRI. Brit J Radiol 2014;87.

18. Hsu LY, Kellman P, Arai AE. Nonlinear myocardial signal intensity correction improves quantification of contrast-enhanced first-pass MR perfusion in humans. Journal of Magnetic Resonance Imaging 2008;27:793-801.

19. Kellman P, Hansen MS, Nielles-Vallespin S et al. Myocardial perfusion cardiovascular magnetic resonance: optimized dual sequence and reconstruction for quantification. J Cardiovasc Magn Reson 2017;19:43.
